# Optical Design of Imaging Spectrometer Based on Linear Variable Filter for Nighttime Light Remote Sensing

**DOI:** 10.3390/s21134313

**Published:** 2021-06-24

**Authors:** Yunqiang Xie, Chunyu Liu, Shuai Liu, Xinghao Fan

**Affiliations:** 1 Changchun Institute of Optics, Fine Mechanics and Physics, Chinese Academy of Sciences, Changchun 130033, China; xieyunqiang131@163.com (Y.X.); liushuai@ciomp.ac.cn (S.L.); ccfanxh@163.com (X.F.); 2 University of Chinese Academy of Sciences, Beijing 100049, China; 3 Key Laboratory of Space-Based Dynamic & Rapid Optical Imaging Technology, Chinese Academy of Sciences, Changchun 130033, China

**Keywords:** nighttime light remote sensing, imaging spectrometer, optical system, high resolution

## Abstract

Nighttime light remote sensing has unique advantages on reflecting human activities, and thus has been used in many fields including estimating population and GDP, analyzing light pollution and monitoring disasters and conflict. However, the existing nighttime light remote sensors have many limitations because they are subject to one or more shortcomings such as coarse spatial resolution, restricted swath width and lack of multi-spectral data. Therefore, we propose an optical system of imaging spectrometer based on linear variable filter. The imaging principle, optical specifications, optical design, imaging performance analysis and tolerance analysis are illustrated. The optical system with a focal length of 100 mm, F-number 4 and 43° field of view in the spectrum range of 400–1000 nm is presented, and excellent image quality is achieved. The system can obtain the multi-spectral images of eight bands with a spatial resolution of 21.5 m and a swath width of 320 km at the altitude of 500 km. Compared with the existing nighttime light remote sensors, our system possesses the advantages of high spatial and high spectral resolution, wide spectrum band and wide swath width simultaneously, greatly making up for the shortage of the present systems. The result of tolerance analysis shows our system satisfy the requirements of fabrication and alignment.

## 1. Introduction

Nighttime light remote sensing can obtain various light information on the earth’s surface at cloudless night, most of which are sent out by artificial lights. In contrast to common remote sensing satellite imageries which are mainly used to monitor the physicochemical characteristics of the earth, nighttime light remote sensing imageries have higher correlation with human activities and possess the advantages of spatiotemporal continuity, independence and objectivity. Therefore, they have been widely used in the field of social science such as gross national product, greenhouse gas emissions, poverty index, urbanization monitoring and evaluation, urban agglomeration evolution analysis and light pollution analysis. In addition to reflecting the lights from urban areas, nighttime light remote sensing can also capture the lights of fishing boats, natural gas combustion, forest fires, etc., so it can also provide important support for many other research fields such as fishery monitoring, major event estimation and natural disaster risk assessment [1,2,3,4,5]. 

Up to now, the Defense Meteorological Satellite Program’s Operational Linescan System (DMSP/OLS) data and the Visible Infrared Imaging Radiometer Suite (VIIRS) instrument onboard the Suomi National Polar Partnership (NPP) satellite are the two most common sources of nighttime light data [6]. The DMSP/OLS has a rich database and can detect the nighttime light radiance from 1.54 × 10^−9^ to 3.17 × 10^−7^ W·cm ^−2^·sr ^−1^ with a swath width of 3000 km [7,8]. However, it has a coarse spatial resolution of 2700 m and can only obtain panchromatic images, limiting its use in fine application [9]. Other shortcomings such as lack of on-board calibration, blooming effects and saturation in urban cores make its application further limited [10]. The NPP-VIIRS has a radiometric range of 3 × 10^−9^ to 2 × 10^−2^ W·cm^−2^·sr^−1^ and swath width of 3000 km [11,12,13]. As the successor to the DMSP/OLS, it has a certain degree of upgrade such as finer resolution of 740 m and on-board radiometric calibration [8,14,15]. However, the resolution is still very low and only panchromatic images can be obtained [16]. Luojia 1–01 satellite launched by Wuhan University in China, has the spatial resolution of 130 m and swath width of 250 km [17]. However, like its predecessors, it cannot capture spectral images [18]. On 9 January 2017, Chang-guang Satellite Technology Co., Ltd. launched the JL1-3B satellite. It provides significant improvements over the previous satellite remote sensors, in terms of increased spatial resolution (0.92 m) and multispectral bands including 430–512 nm, 489–585 nm and 580–720 nm [19]. However, its swath width is only 11 km, and the number of wavebands is too few to provide more detailed information. Imaging information with high spatial resolution and high spectral resolution in a wide waveband is imperative to identify the type of the light source and their spatial arrangement [20]. Since the light from different areas such as residential, commercial and war area shows different spatial and spectral characteristics, high-resolution datasets have unique advantages over the coarse resolution imagery in monitoring, analyzing, evaluating, and predicting the changes on the earth’s surface and thus contribute to quantify and track the dynamic of human activities and their impacts on environment and economy [21,22]. In sum, a nighttime light remote sensor that can capture imageries with high spectral and spatial resolution in a wide waveband and swath width is worth studying. 

In this paper, we present an optical system of imaging spectrometer based on linear variable filter for nighttime light remote sensing that can obtain the multi-spectral images of eight spectral bands in the range of 400–1000 nm with a spatial resolution of 21.5 m and a swath width of 320 km. Compared with the imaging spectrometers based on prism, prism-grating and prism-grating-prism dispersion module which consist of two optical system namely, the telescopic system and the spectroscopic system, our system possess the advantages of compactness, light weight, and low cost, although the spectral resolution is lower. For example, the imaging spectrometer based on prism-grating-prism developed by Xue consists of telescope system and spectral imaging system [23]. The imaging spectrometer contains 24 lenses and has a total length of 300 mm. The imaging spectrometer based on prism-grating by Chen contains 22 lenses [24]. In contrast, our system contains 15 lenses, and the total lenses is 250 mm. Although spectral shift may occur to our spectrometer due to the inherent limitation of linear variable filter, it can be greatly alleviated by spectral calibration. Compared with the existing nighttime light remote sensors, our system meeting the requirements of high spatial and high spectral resolution, wide working band and wide swath width simultaneously, greatly making up for the shortage of the present systems. For an imaging spectrometer for nighttime light remote sensing, it is necessary to maintain illumination uniformity of imaging plane to ensure sufficient SNR [25,26,27]. By analyzing the energy distribution of the focal plane and pupil aberration, we apply to the pupil coma to improve the illumination of the edge field of view. Since the waveband of our system is too wide, it is essential to correct the chromatic aberration and secondary spectrum to get excellent image quality. In order to obtain an apochromatic system, some special materials such as crystals and liquids are usually used [28,29]. However, the disadvantages of these materials such as fragility, instability and poor availability prevent their applications in aerospace. Fortunately, the Buchdahl dispersion model can help to provide an effective method to select glass materials for apochromatic systems [30]. In the design process of our system, the chromatic aberration and secondary spectrum are corrected by properly choosing glass for certain lenses with the dispersion vector method based on the Buchdahl dispersion model. Other aberrations such as astigmatism and distortion are reduced by adjusting the position of aperture stop and shape and bending factors of certain lenses. After iterative optimization in software ZEMAX, we get the final optical system with excellent image quality. We have carried out the tolerance analysis and the result shows that the system is qualified for practical application.

## 2. Imaging Principle

**Figure 1:**
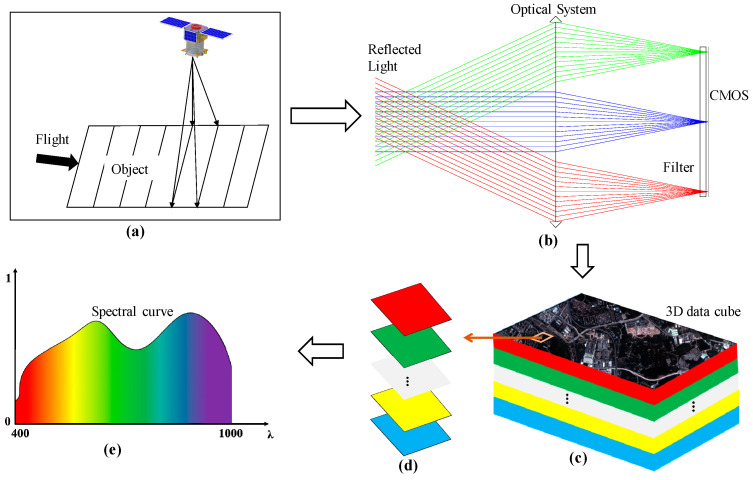
Schematic diagram of the imaging spectrometer’s optical system and its push-broom imaging principle. (**a**) Push-broom imaging principle, (**b**) Structure of the imaging spectrometer, (**c**) Three-dimensional data cube, (**d**) Data column in the data cube, (**e**) Spectrum curve of the object.

A schematic diagram of the imaging spectrometer’s optical system and its imaging principle is shown in Figure 1. The system consists of an optical system and a filter which is placed in proximity to the CMOS, with the coated side towards the pixels. The imaging spectrometer is a push-broom system and the working principle of this type of system is shown in Figure 1a. The light from the ground target is imaged by the optical system. After passing through the filter, the light forms an image on the focal plane and is finally received by the CMOS, as shown in Figure 1b, where the lights with different color represent different fields of view. We can therefore obtain multiple linear images of different wavebands which can be expanded into plane images using the push-broom imaging mode of the spectrometer.

The data captured by the imaging spectrometer is referred to as a three-dimensional data cube denoted by two-dimensional space and one-dimensional spectrum [31]. The data cube contains spectral and spatial information of the targets within the whole scanning range of the imaging spectrometer, as shown in Figure 1c. Each target in the scanning range corresponds to a column in the data cube which can be converted into a spectral curve for spectrum characteristics analysis, as shown in Figure 1d,e. 

## 3. Optical Specifications

The imaging spectrometer is mounted on a low-orbit satellite at the altitude of 500 km and the technical index requires that the ground sample distance (GSD) is less than 21.5 m. Such sampling frequency is sufficient to distinguish the adjacent street lamps on most roads [32]. Besides, it can help to distinguish large buildings by detecting all the artificial light on them such as billboards and neon lights [33]. The focal length of the optical system $f^{\prime }$ can be given by

(1) $$f^{\prime }=\frac{\alpha \cdot H}{GSD}$$

where α is the size of the CMOS pixel, *H* is the detection distance. The CMOS image sensor GSENSE5130 produced by Gpixel Inc with 5056 × 2968 pixels and 4.25 μm × 4.25 μm pixel size is chosen as the detector of our system [34]. It is calculated that the focal length is 98.8 mm. In order to have abundant allowance, we choose 100 mm as the focal length of our system. It should be noted that the ground sample distance of 21.5 m corresponds to the nadir pixels. For the marginal pixels, the corresponding ground sample distance will be slightly larger.

The width coverage *L* of our imaging spectrometer is required to be more than 320 km to be sufficient to cover two cities during one shot. Besides, such a wide coverage can reduce the number of revisiting when capture a large area. It can be calculated by the following equation that the width of the image plane *l* is 64 mm.

(2) $$l=\frac{f^{\prime }\cdot L}{H}$$

**Figure 2:**
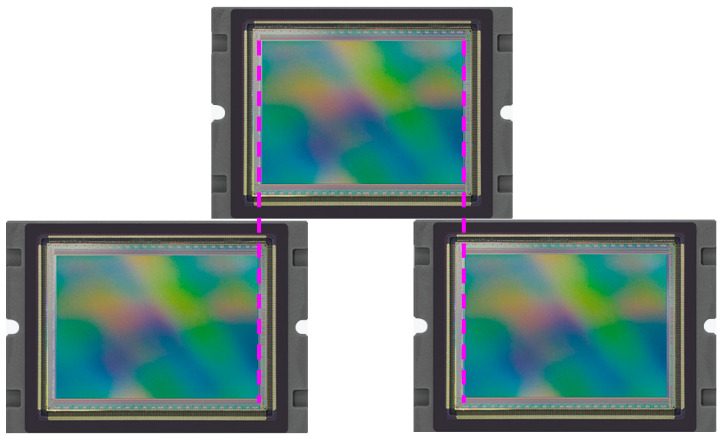
Schematic diagram of image plane spliced by three image sensors.

Since the width of the image sensor GSENSE5130 is 21.488 mm, less than that of the image plane, we use three image sensors to splice the image plane in the form of Figure 2. In order to avoid the chink between adjacent sensors in the assembly process, an overlap area of 50 pixels will be set between them. In this case, the width coverage can be calculated as 320.2 km by

(3) $$L=GSD_{\min}\cdot \left(P_{1}+P_{2}+P_{3}\right)$$

where, *P*_1_ = 5006, $P_{2}=5056$ and $P_{3}=5006$ which are the effective number of pixels of the three sensors, $GSD_{\min}$ is the ground sample distance calculated by Equation (1) when the focal length is 100 mm.

Through geometry calculation, we can get that the circumcircle diameter of the spliced image plane is 77 mm. Then the half-field angle ω of the optical system can be calculated as follows:

(4) $$\omega =\arctan\frac{d}{2f^{\prime }}=21.06^{\circ }$$

where d is the circumcircle diameter of the spliced image plane. We set ω to 21.5° to have abundant allowance.

**Figure 3:**
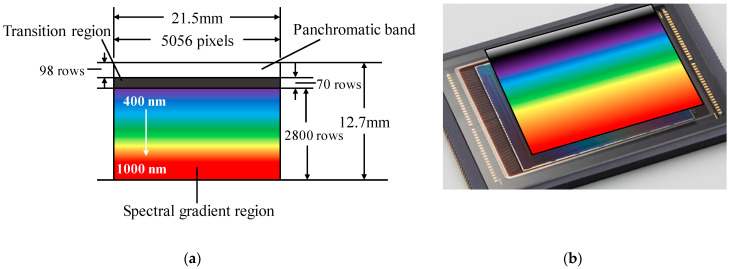
(**a**) The corresponding relationship between filter and detector. (**b**) schematic of the structure.

In order to match the detector in size, the size of the linear variable filter we choose is 33.2 mm × 33.2 mm × 1.2 mm and the effective coating area is 21.5 mm × 12.7 mm. The corresponding relationship between the filter and the detector pixels is shown in Figure 3. The area of the panchromatic band corresponds to the first 98 rows pixels of the sensor and the size is about 0.42 mm × 12.7 mm. The next 70 rows of pixels correspond to the transition region of the filter whose size is about 0.3 mm × 12.7 mm. The remaining area of the filter corresponding to the next 2800 rows of pixels is the spectral gradient region in the range of 400–1000 nm. The central wavelength varies along the longitudinal direction of the filter. The full width half maximum (FWHM) of filter is around 5% of the center wavelength and the transmittance is above 90%. Accurate co-registration of bands and different rows of pixels will be realized by spectral calibration. After co-registration of bands and detectors, spectral images can be obtained by integrating the data of corresponding pixels in the push-broom mode. The system can obtain eight spectral images and one panchromatic image at one time and thus can achieve accurate detection [35]. The bandwidth of spectral image can be set between 20 nm and 40 nm according to practical need. However, since the FWHM of the filter increases with the central wavelength increasing, the spectral images in the waveband near 1000 nm will have wider bandwidth. It should be noted that the spectral bands can be reset on orbit because of the use of the linear variable filter.

In order to ensure the resolution and accuracy of measurement, the modulation transfer function (MTF) of the system should be greater than 0.12 at the Nyquist frequency which can be calculated as 118 lp/mm by

(5) $$N=\frac{1000}{2\cdot \alpha }$$

where *N* is the Nyquist frequency, α is the size of the CMOS pixel. The MTF can be calculated as follows [36,37]:

(6) $$\mathrm{MTF}={\mathrm{MTF}}_{\mathrm{design}}\cdot {\mathrm{MTF}}_{\mathrm{man}}\cdot {\mathrm{MTF}}_{\mathrm{sample}}$$

where ${\mathrm{MTF}}_{\mathrm{design}}$ is the MTF of design value of the optical system, ${\mathrm{MTF}}_{\mathrm{man}}$ is the MTF of optical system manufacturing which can be set as 0.8 based on engineering experience, ${\mathrm{MTF}}_{\mathrm{sample}}$ is the MTF of detector whose value is 0.5. After calculation, ${\mathrm{MTF}}_{\mathrm{design}}$ should be greater than 0.3.

High signal-to-noise ratio (SNR) of imaging spectrometer is necessary for nighttime light remote sensing, as it enables system to distinguish the target from the background. The SNR [38] is defined as the ratio of signal power to noise power, which can be calculated by

(7) $$SNR=\frac{S_{e}}{N_{e}}=\frac{S_{e}}{\sqrt{S_{e}+\sigma_{R}^{2}+D_{e}}}$$

where *S_e_* is the number of signal electronics, $N_{e}$ is the number of noise electronics, $\sigma_{R}=1.6\;e^{-1}$, is the readout noise, $D_{e}=3\;e^{-1}/s/\mathrm{pix}$, is the dark current. 

The number of signal electrons within the integrated period is obtained by:

(8) $$S_{e}=\frac{\pi A_{d}Mt_{\mathrm{int}}}{4F^{2}h\nu }L_{0}\tau_{0}\eta$$

where $A_{d}$ is the area of a single pixel, M is the integration number of our CMOS whose range is from 1 to 186, $t_{\mathrm{int}}$ is the integration time of the system, $L_{0}$ is the radiance at the entrance pupil, which can be obtained by the software MODTRAN, $\tau_{0}$ is the transmittance of the optical system. We expect to use 15 lenses made of optical glass with a transmittance of 0.995 when the thickness is 10 mm in the spectral range of 500–1000 nm. The transmittance in the range of 400–500 nm will be lower; we must improve the quality of corresponding images by the increase in the integration number or specific image processing algorithm. All surfaces of the lenses will be coated with the anti-reflective film which guarantees a transmittance of more than 0.995 of each surface. Since the average transmittance of the linear variable filter is 0.9, the transmittance of the optical system can be calculated as 0.718. We set $\tau_{0}$ as 0.7 to have abundant allowance. η is the quantum efficiency of the detector, F is the F-number of the optical system, h is Plank’s constant, v is the frequency. The values of the variables used to calculate the SNR are listed as below: $A_{d}=4.25\mu m\times 4.25\mu m$, $t_{\mathrm{int}}=0.003s$, η=65%.

**Figure 4:**
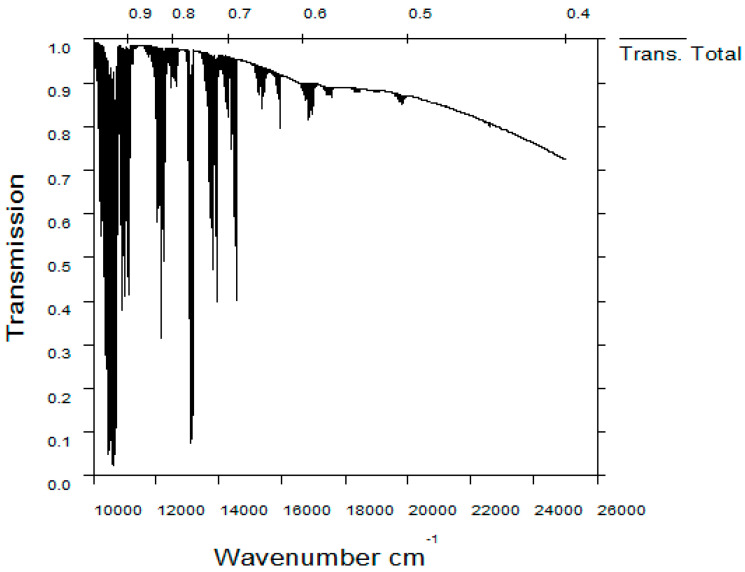
The atmospheric transmittance per wavelength in the spectral range of 400–1000 nm.

The technical index requires that the SNR of the system is more than 10 when the radiance of the target is 1E-7 W/cm^2^/sr in each imaging spectral band to distinguish most human activities [22]. The atmospheric transmittance per wavelength in the spectral range of 400–1000 nm obtained from the software MODTRAN is shown in Figure 4. The main parameters used to calculate the transmittance are shown in Table 1. Ignore the band with extremely low transmittance, we take the average transmittance as 85%. According to Figure 4, the wavelength range 400 nm to 450 nm should also be ignored when the radiance of object is quite low due to the poor atmospheric transmittance. Calculated by the above formulas, when F-number and integration number are 4 and 86, respectively, the SNR is 10. When the integration number is 86, the stability of the system should be controlled within 0.001 degree, which is feasible to the existing satellite. Besides, image motion compensation needs to be applied to avoid the image blurring caused by the relative motion between the object and detector. Spatial calibration is also necessary to achieve high-precision detection. In order to balance the performance and volume and weight of the system, we set the F-number to 4. The technical specifications of the optical system are listed in Table 2.

## 4. Image Plane Illumination Analysis

Relative illumination is the ratio of the illumination of the edge of the image plane to that of the center point. Nighttime light remote sensing require that the relative illumination of image plane should be greater than 80% for all fields of view to ensure sufficient SNR. However, the image plane illumination decreases as the field of view increases. Since the half field of view of our system is 21.5°, it is necessary to maintain the illumination uniformity of the image plane. In order to take effective measures to solve this problem, it is essential to determine the relation between the image-plane illumination and other parameters of optical system.

**Figure 5:**
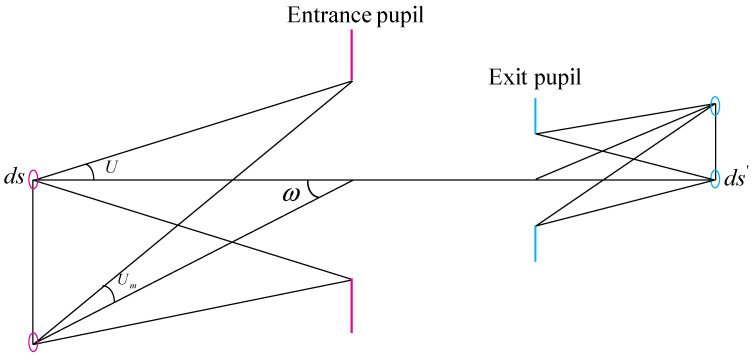
Transfer relation between object and image in optical system.

Figure 5 shows the transfer relation between object and image in optical system. Here ds is a radiation element on the object plane, which is imaged on the image plane as $ds^{\prime }$ by the optical system. The radiation flux [39] received by the system from ds is

(9) $$\Phi =\pi L\cdot \sin^{2}U\cdot ds$$

where L is the radiance brightness of the object, U is the aperture angle in object space. If the transmittance of the system is τ, the radiation flux of $ds^{\prime }$ is

(10) $$\Phi^{\prime }=\Phi \cdot \tau$$

Thus, the illumination of $ds^{\prime }$ is

(11) $$E^{\prime }=\frac{\pi \tau L\cdot \sin^{2}U\cdot ds}{ds^{\prime }}=\frac{\pi \tau L\cdot \sin^{2}U}{\beta^{2}}$$

where β is the lateral magnification of the optical system.

For the off-axis field of view, according to the geometric principle, the relationship between on-axis and off-axis aperture angles can be obtained as

(12) $$\sin^{2}U_{m}=\sin^{2}U\cos^{4}\omega$$

Thus, the illumination of off-axis point on image plane is

(13) $$E^{\prime }=\frac{\pi \tau L\cdot \sin^{2}U\cdot \cos^{4}\omega }{\beta^{2}}$$

As analyzed above, the illumination is proportional to the fourth power of the cosine of the angle of half field of view. Therefore, for the optical system with large field of view, the illumination at the edge of image plane is much lower than that at the center position, and we have to adjust the system to improve the uniformity of illumination on image plane.

According to Equation (10), when the half field of view increases, in order to keep the detection ability of the system unchanged, we can only reduce the lateral magnification to improve the illumination. Barrel distortion is the kind of aberration that compresses the edge of the image plane and thus it can make the lateral magnification decrease with the increase in the field of view. The barrel distortion could then be employed to improve the illumination of the edge field of view. However, for an imaging spectrometer based on linear variable filter, too much distortion will lead to sever spectrum crosstalk between different optical channels. Thus, taking the use of barrel distortion to promote the uniformity of image plane illumination is not desirable, and we have to find other ways.

Unlike paraxial optical system, pupil aberrations increase sharply in the optical system with large field of view [40]. Pupil aberrations in the optical system with large field of view will make the location and size of the entrance pupil at the off-axis field of view different from that at the paraxial field of view. Among several pupil aberrations, pupil spherical aberration and pupil coma have the greatest influence on the entrance pupil. Pupil spherical aberration makes the location of the entrance pupil at the edge of the field of view deviate from the ideal position, thus it mainly affects the calibration of the chief ray and has less influence on the relative illumination. Pupil coma can make the beam width of the off-axis field at the entrance pupil larger than that of the center field when both of them completely fill the stop aperture [41]. Such vignetting effect of pupil aberration can ensure that the off-axis object has a larger aperture than the on-axis object, so it is very beneficial to improve the relative illumination. Thus, we will improve the image plane illumination of the edge field of view by taking advantage of pupil coma.

## 5. Optical System Design and Optimization

### 5.1. Initial Optical Structure Design

According to the technical specifications of our system calculated in Section 3, we selected an inverse telephoto structure with 13 lenses as the starting point for further design. The first element of the system is set as a negative meniscus lens to compress the angle of the light in the off-axis field of view relative to the optical axis to reduce the diameter of the other elements. Besides, taking a negative meniscus lens as the first element contributes to correct chromatic aberration and generate pupil coma which is helpful to improve the image plane relative illumination. Due to the potential for spectral shift of the linear variable filter, the system should possess a telecentric optical path in image space to ensure that the chief ray in each field of view can be perpendicular incident on the filter and focal plane. In order to realize the telecentric characteristic of the system, a positive lens group is arranged in front of the focal plane to compress the emergent light so that it is parallel to the optical axis. two separated positive and negative lens groups are introduced to the system to emend the field curvature. Since the working band of the system is too wide, it is important to eliminate chromatic aberration and secondary spectrum. In order to achieve excellent chromatic correction, cemented doublet composed of a low dispersion crown glass and a short flint glass is usually used. However, cemented doublet is structurally unstable and rarely used in aerospace camera. Thus, we choose two air-spaced doublets that present the same optical properties in the middle of the system for apochromatism. In order to increase the transmittance of the system, we chose the glass with the high transmittance in the range of 400–1000 nm and controlled the angle between the light and the normal of each surface as small as possible to reduce the reflection of the light. For the purpose of increasing the optimization degrees of freedom, we added two lenses to the system.

**Figure 6:**
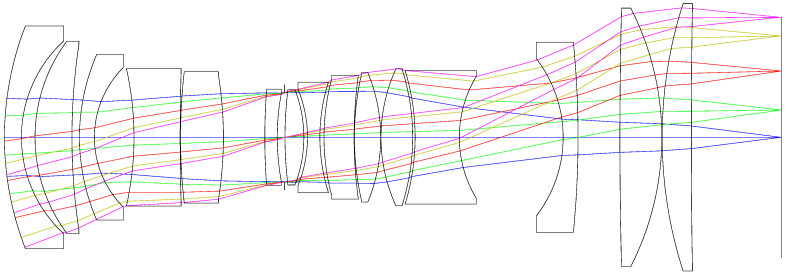
Optical configuration of the initial system.

**Figure 7:**
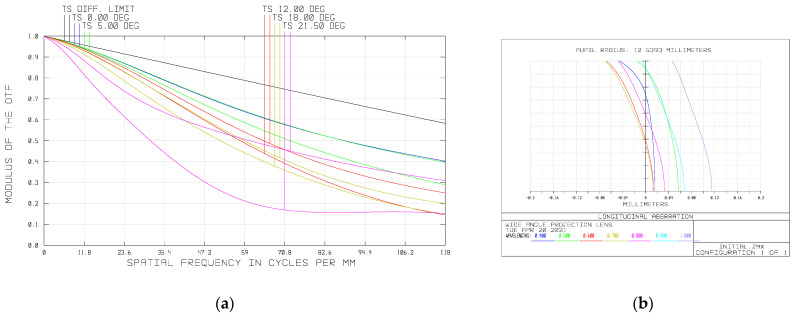
(**a**) MTF; (**b**) longitudinal aberration of the initial system.

We then optimized the system with optical design software ZEMAX. After several iterations of optimization, the initial optical system was obtained, as shown in Figure 6. This is a quasi-symmetric system with a telecentric light path in image space. The MTF and longitudinal aberration of the system is shown in Figure 7. It can be seen that the MTF of the marginal field of view is very low, which means a poor image quality. We take the chromatic aberration at the pupil of 0.707 as the major reference, because achromatism of such pupil makes the chromatic aberration at the full pupil close to that of the paraxial area and thus achieve the best achromatic effect. In the figure of the longitudinal aberration curves, the wavelengths of 400 nm, 700 nm, 1000 nm do not converge in a point at the pupil of 0.707 and the chromatic aberration of the system at this zone is 0.0718 mm, which means that the system does not achieve apochromatism. To enhance the apochromatic ability, we need to replace the inappropriate glass in the system. Therefore, material selection method is the key to the subsequent design. In this paper, we will use the Buchdahl model to analyze the dispersion characteristics of materials and select proper glass for our system.

### 5.2. Buchdahl Dispersion Model

Dispersion is defined as the variation of refractive index with wavelength, and thus accurate description of the refractive index of glass materials is the premise of studying dispersion. The most commonly used model to calculate the refractive index of glass materials is

(14) $$N^{2}\left(\lambda \right)=A_{0}+A_{1}\cdot \lambda^{2}+A_{2}\lambda^{-2}+A_{3}\lambda^{-4}+A_{4}\cdot \lambda^{-6}+A_{5}\lambda^{-8}$$

where N(λ) is the refractive index when the wavelength is λ, $A_{i}$ is the coefficients of glass material.

The dispersive properties of glass can be modeled using the Taylor series expansion, which is expressed as

(15) $$\Delta N\left(\lambda \right)=a_{0}+a_{1}\cdot \Delta \lambda +a_{2}\cdot {\left(\Delta \lambda \right)}^{2}+a_{3}\cdot {\left(\Delta \lambda \right)}^{3}+\ldots$$

where $\Delta N\left(\lambda \right)=N\left(\lambda \right)-N\left(\lambda_{0}\right)$, $\Delta \lambda =\lambda -\lambda_{0}$, $\lambda_{0}$ represents the base wavelength. It can be seen that Equation (11) describes the relationship between the square of refractive index and wavelength, which will cause the calculation process of Equation (12) very complicated. Besides, Equation (12) converges very slowly even for a small value of Δλ, leading to a large number of terms to be calculated to obtain accurate result [42]. For the theoretical analysis of dispersive properties, Equations (11) and (12) are too cumbersome for practical application. In order to simplify the calculation process and ensure the accuracy, fast convergent formula of refractive index is essential.

Buchdahl model [43] simplifies the dispersion equation by changing the wavelength λ into chromatic coordinate ω, which is defined as

(16) $$\omega =\frac{\lambda -\lambda_{0}}{1+\alpha \left(\lambda -\lambda_{0}\right)}$$

where α is a constant that makes Buchdahl refractive index equation converge fast. It can be calculated by

(17) $$\alpha =\frac{1}{\lambda_{0}-\lambda^{*}}$$

Here the unit of $\lambda_{0}$ is μm. $\lambda^{*}=0.174\;\mu m$, which is the Hartman formula constant and can be used to model for all types of glass [44]

By replacing the wavelength with chromatic coordinate, the Buchdahl’s equation for the refractive index is given by

(18) $$N=N_{0}+v_{1}\omega +v_{2}\omega^{2}+\ldots +v_{i}\omega^{i}$$

where $v_{i}$ is the Buchdahl dispersion coefficients, which characterize the dispersion of the glass and the value is unique to each glass. Buchdahl’s equation for the refractive index converges very fast. When used to model the dispersive properties of the glasses, a quadratic equation is sufficient to achieve satisfactory accuracy. Thus, only $v_{1}$ and $v_{2}$ are necessary to be determined and the calculation process can be found in reference [43].

### 5.3. Method of Glass Selection for Apochromatic

As a function of wavelength, dispersion power [45] of an optical glass is defined as

(19) $$D\left(\lambda \right)=\frac{\Delta N\left(\lambda \right)}{N_{0}-1}$$

Through proper variables’ transformation, the dispersion power can be expressed by Buchdahl model as

(20) $$\left\{ \begin{array}{l} D\left(\lambda \right)=\frac{\Delta N}{N_{0}-1}=\sum_{i=1}^{n}\eta_{i}\cdot \omega^{i}\\ \eta_{i}=\frac{v_{i}}{N_{0}-1} \end{array}\right.$$

where $\eta_{i}$ is called the dispersion coefficient, *n* is the order of the equation.

The expression for the lateral chromatic aberration of an optical system with the object at infinity can be expressed by Buchdahl model as

(21) $$\mathrm{TAchc}=-y_{1}\cdot D_{0}=-y_{1}\cdot \sum_{i=1}^{m}\frac{y_{i}^{2}\cdot \phi_{i}}{y_{1}^{2}\cdot \phi_{0}}\cdot D_{i}\left(\lambda \right)$$

where TAchc is the lateral chromatic aberration, $D_{0}$ is the dispersion power of the whole system, $y_{i}$ is the ray height of the i-th lens, $\phi_{i}$ is the focal power of the i-th lens, $\phi_{0}$ is the focal power of the whole system, m is the number of lenses in the optical system. Taking the quadratic Buchdahl model into Equation (20), we can get the following equations:

(22) $$\left\{ \begin{array}{l} D_{0}=\eta_{10}\omega +\eta_{20}\omega =\sum_{i=1}^{m}\alpha_{i}\cdot \left(\eta_{1i}\cdot \omega +\eta_{2i}\cdot \omega \right)\\ \alpha_{i}=\frac{y_{i}^{2}\cdot \phi_{i}}{y_{1}^{2}\cdot \phi_{0}}\\ \eta_{j0}=\sum_{j=1}^{m}\alpha_{i}\eta_{ji}\left(j=1,2\right) \end{array}\right.$$

where $\eta_{10}$ and $\eta_{20}$ are the primary and secondary dispersion coefficients of the whole system, $\eta_{1i}$ and $\eta_{2i}$ are primary and secondary dispersion coefficients of the i-th lens, $\alpha_{i}$ is scale factor. If we take the dispersion properties of each glass as a vector $\left(\eta_{1i},\eta_{2i}\right)$ in the coordinate system denoted by $\eta_{1}$ and $\eta_{2}$, then the vector $\left(\eta_{10},\eta_{20}\right)$ representing the dispersion of the whole system can be calculated by

(23) $${\vec{G}}_{0}=\sum_{i=1}^{m}\alpha_{i}{\vec{G}}_{i}$$

where $\vec{G_{0}}=\left(\eta_{10},\eta_{20}\right)$, $\vec{G_{i}}=\left(\eta_{1i},\eta_{2i}\right)$. We can then take the module of $\vec{G_{0}}$ as the criterion to measure the apochromatic ability of the optical system. In order to obtain an apochromatic optical system, we should adjust the scale factors and change the glass in certain lenses to shorten the module of the vector $\vec{G_{0}}$.

### 5.4. Dispersion Vector Analysis and Glass Replacement

**Figure 8:**
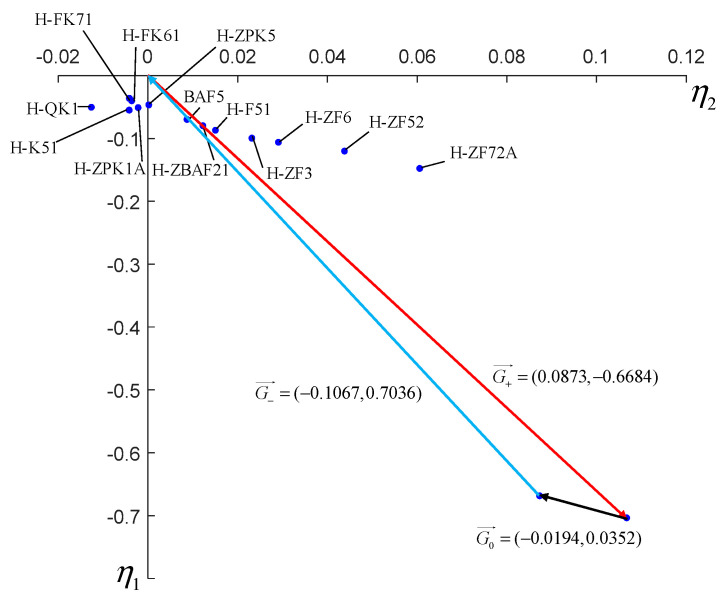
Dispersion coefficient of each lens and the dispersion vector of the initial system.

**Figure 9:**
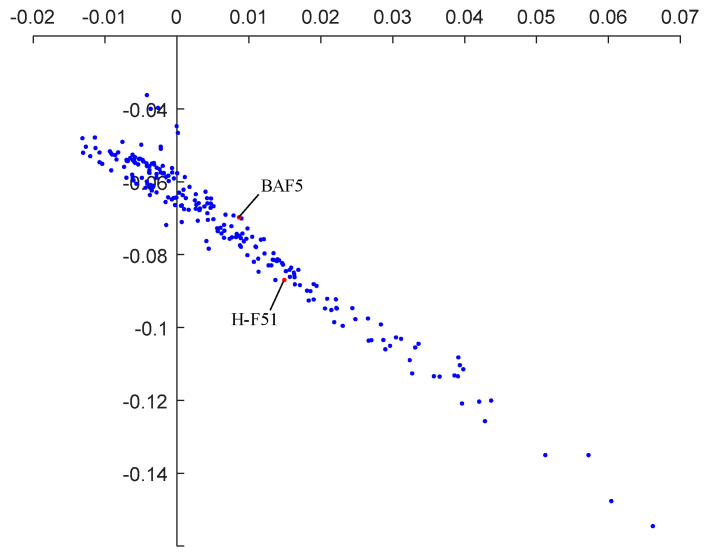
Dispersion coefficient of each lens and the dispersion vector of the initial system.

**Figure 10:**
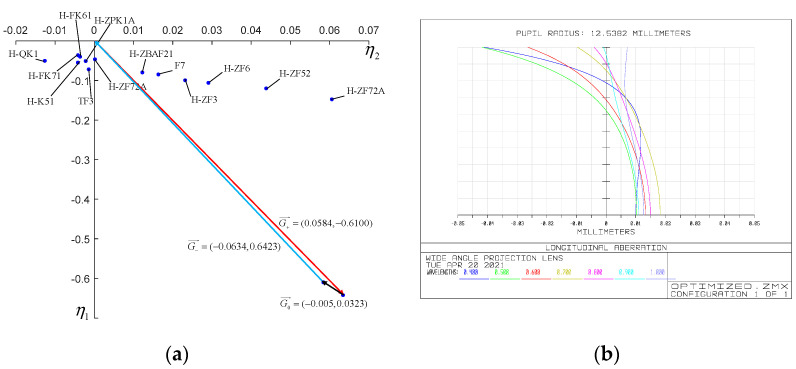
(**a**) Dispersion coefficient of each lens and the dispersion vector of the optimized system; (**b**) Longitudinal aberration curves of the optimized system.

We have conducted raytracing to our initial optical system and the focal power, ray height, refractive index at the reference wavelength, scale factor of dispersion vector and dispersion coefficient of each lens are listed in Table 3. The coordinate of the dispersion coefficient of each lens and the dispersion vector of the whole system in the two-dimensional coordinate system $\left(\eta_{1},\eta_{2}\right)$ are shown in Figure 6. The dispersion vector of the whole system is $\vec{G_{0}}=\left(-0.0194,0.0352\right)$ and its module is 0.0402. It can be seen from Figure 8 that the sum dispersion vector of the negative lenses is far away from that of the positive lenses, which lead to a large module of $\vec{G_{0}}$ and thus an excessive chromatic aberration. In order to reduce the module of $\vec{G_{0}}$, we need to replace the glass material of some lenses. In consideration of the need to maintain the stability of the focal power, the glass far from the origin in the dispersion vector coordinate system cannot be replaced. However, the glass close to the origin has little effect on the chromatic aberration of the system. Thus, we should change the glass in the middle of Figure 8. It can be seen that BAF5, H-ZBAF21, H-F51 and H-ZF3 are in the middle place of Figure 8. However, H-ZF3 and H-ZBAF21 correspond to the second lens and the fifteenth lens of the system, respectively. In our system, the second lens is used to cooperate with the first lens to compress the incident light and reduce the primary aberration and thus has a great influence on the light path. Therefore, the material of the second lens cannot be replaced. As we mentioned in Section 5.1, the fifteenth lens is arranged to compress the emergent light to realize the telecentric characteristic of the system. If the material of the fifteenth lens is changed, the light path will be affected, leading to the loss of telecentric characteristic. BAF5 and H-F51 correspond to the eighth and twelfth lenses, and they are all part of the air-spaced doublets used to reduce chromatic aberration, as mentioned in Section 5.1. Therefore, we choose to replace the glass materials of the eighth and twelfth lenses. The new glass we select should be close to the original ones in the coordinate system $\left(\eta_{1},\eta_{2}\right)$ so that their coefficients do not differ too much and thus prevent the increase of the other aberrations and excessive change of focal power. Figure 9 shows the dispersion coefficient of all glasses produced by CDGM. According to the principles above, glass materials TF3 and F7 are selected to replace BAF5 and H-F51. After the materials’ replacement, we optimize the system with the software ZEMAX and the raytracing parameters of the optimized system are listed in Table 4. The dispersion vector of the optimized optical system is shown in Figure 10a, from which we can see that the module of the vector $\vec{G_{0}}$ is 0.0326, much smaller than the former system. The longitudinal aberration curves of the system are shown in Figure 10b. It can be seen that the wavelengths of 400 nm, 700 nm, 1000 nm converge in a point at the pupil of 0.707. The chromatic aberration and secondary spectrum of the system at the pupil of 0.707 is 0.06 μm and 0.1 μm, respectively, much less than that of the initial system. Thus, the system actualizes the apochromatism.

### 5.5. Final System

**Figure 11:**
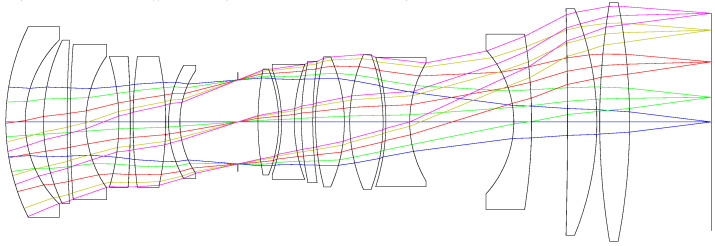
Optical layout of the final system.

The secondary spectrum is only related to the focal length of the whole system and relative partial dispersion and Abbe number of the glass. Therefore, when the focal length and the glass in the system are fixed, the secondary spectrum will not increase when optimizing the structure parameters of the system. The position of the stop aperture plays an important role in determining the lens size and the correction of distortion and field curvature, and thus, we have to adjust it for a better image quality. Besides, we should also alter the shape and bending factors of the lenses to control the distortion of the system at a low level. There must be enough distance between different elements to provide adequate clearance for mechanical spacer and to correct the coma and astigmatism. In order to improve the optical transmittance of the system, all surfaces of the lenses will be coated with the anti-reflective film. The thickness of each lens is controlled within a reasonable range to reduce the weight and improve machinability. We then optimized the system using the software ZEMAX after setting the operands to constrain the geometric parameters and optical characteristics. The total axial length of the final system is about 249 mm, and the weight is 1.03 kg. The transmittance of the final system (including linear variable filter) is 0.723 and 0.463 in the spectral range of 500–1000 nm and 400–500 nm, respectively. As mentioned in Section 3, we will improve the quality of images in the range of 400–500 nm by the increase of the integration number or specific image processing algorithm. The optical layout is shown in Figure 11.

## 6. Image Performance and Tolerance Analysis

### 6.1. Image Performance

**Figure 12:**
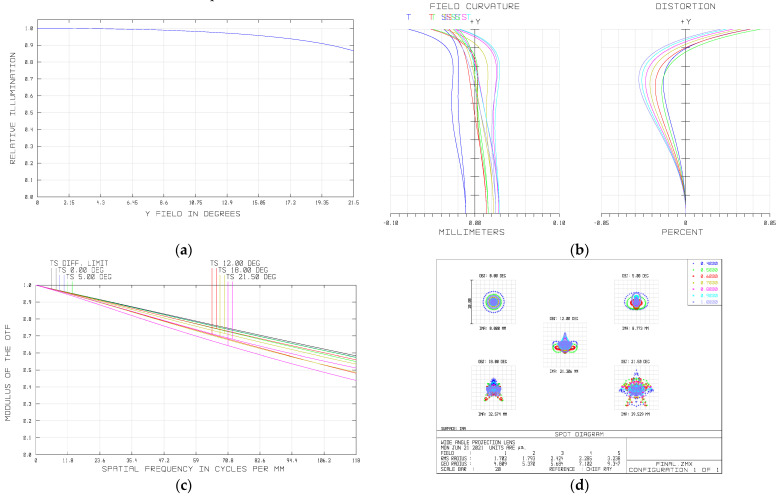
(**a**) Relative illumination of the image plane; (**b**) Field curvature and distortion of the final system; (**c**) MTF of the final system; (**d**) Spot diagram of the final system.

We have known from Section 4 that the relative illumination of the image plane decrease with the fourth power of the cosine value of the half field of view. Our system has a large field of view, and the illumination uniformity must be taken into account. As analyzed in Section 4, we apply to the pupil coma to improve the illumination of the off-axis field of view. The relative illumination curve is shown in Figure 12a, and it can be seen that the relative illumination of the edge field of view is above 85%, better than the design requirement. 

As an imaging spectrometer, the distortion must be controlled within a reasonable range. Figure 12b shows the curves of the distortion and field curvature of the system, from which we can see that the maximum distortion is less than 0.05% and the distortion correction is excellent, ensuring high-precision detection of the system. The field curvature of all bands is less than 0.08 mm, meaning a good correction.

The MTF represents the image quality of the optical system. As is shown in Figure 12c, the MTF of the full field of view exceeds 0.4 at 118 lp/mm, better than the design requirement. Therefore, the system possesses a good contrast ratio and the image quality of on axis and off axis exhibits good consistency. The spot diagram is shown in Figure 12d. It can be seen that the root mean square (RMS) diameter is less than on single pixel size (4.25 μm) over the whole field of view, meaning a high resolution of the system.

### 6.2. Tolerance Analysis

Tolerance analysis is used to systematically analyze the influence of slight error or chromatic dispersion on optical system performance. The purpose of tolerance analysis is to determine the types and values of the errors and introduce them into the optical system to analyze whether the system performance meets the practical requirement. In our system, we take the average diffraction MTF at 118 lp/mm as the evaluation criterion and allocate the tolerance as Table 5. According to the result of tolerance analysis, the average diffraction MTF at 118 lp/mm is above a value of 0.26 with a probability of 90%, which means that the system meets the requirement of practical fabrication and alignment.

## 7. Discussion and Conclusions

We have designed an apochromatic optical system with a wide field of view for spectral remote sensing of nighttime light. Compared with the existing nighttime light remote sensors, our system meeting the requirements of high spatial and high spectral resolution, wide working band and wide swath width simultaneously. It can obtain the spectral information of eight bands in the range of 400 nm to 1000 nm with the ground sample distance of 21.5 m while the radiance of the target is above 1E-7 W/cm^2^/sr. Besides, the width coverage of our system reaches 320 km. We first determined the parameters of the system according to the practical requirement. Then we designed the initial structure of the optical system. In the optimization process, we applied the pupil coma to improve the relative illumination of the edge field of view. In order to achieve apochromatism, we employed the vector operation based on Buchdahl model to analyze the dispersion characteristics of glass and replace the glass materials of certain lenses in the system. After the final optimization, the system possesses excellent image quality. The RMS spot sizes of different field of view are all less than 4.25 μm, within one-pixel size. The MTF is above 0.4 at the Nyquist frequency of 118 lp/mm. The field curvature and distortion are also well corrected. The result of tolerance analysis shows that the system meets the requirement of fabrication and alignment.

The scheme of combining three lenses together can also enlarge the field of view. Such scheme can simplify the process of optical design. However, the volume and weight of the whole system will increase significantly, and the higher stability of the platform will be required. In contrast, our single lens scheme possesses the advantages of compactness and lightweight. Besides, it needs less balancing weight, promoting the stability of the whole system. Another scheme is to introduce aspheric surface to the optical system to use less glass and thus improve the transmittance. However, considering the price of an aspheric lens is about ten times that of spherical lens, the cost will increase sharply. The reflective optical system has a transmittance close to 1 but has a high cost and is difficult to realize the telecentric characteristics.

## Figures and Tables

**Table 1:** Main parameters of MODTRAN used to calculate transmittance.

Parameters	Value
Model Atmosphere	Middle altitude summer
Water vapor column	1.0 gm/cm^2^
CO2 Mixing Ratio	330.0 ppmv
Aerosol model used	Rural –VIS-23km

**Table 2:** Technical Specifications of the optical system.

Parameters	Value
Wavelength range	400–1000 nm
Focal length	100 mm
F-number	4
Field of view	±21.5°
MTF	>0.3@118lp/mm
Distortion	<0.05%

**Table 3:** Ray-tracing parameters of the initial system.

	**1**	**2**	**3**	**4**	**5**	**6**		**7**
**Glass**	**H-K51**	**H-ZF3**	**H-FK71**	**H-ZF6**	**H-ZBAF21**	**H-ZF6**		**H-ZPK1A**
$\varphi_{j}$	−0.006834	0.01047	−0.00721	−0.008528	0.009377	−0.006079		0.0192
$Y_{j}$	12.539	12.636	11.958	11.936	13.251	14.274		14.412
$N_{0}$	1.5190	1.7069	1.4542	1.7435	1.7151	1.7435		1.6135
$\alpha_{j}$	−0.6856	1.0666	−0.6578	−0.7752	1.0505	−0.7902		2.5444
$\eta_{1}$	−0.0549	−0.0995	−0.0362	−0.1060	−0.0797	−0.1060		−0.0509
$\eta_{2}$	−0.0042	0.0232	−0.0041	0.0291	0.0122	0.0291		−0.0022
	**8**	**9**	**10**	**11**	**12**	**13**	**14**	**15**
**Glass**	**BAF5**	**H-ZF72A**	**H-FK61**	**H-ZPK5**	**H-F51**	**H-QK1**	**H-ZF52**	**H-ZBAF21**
$\varphi_{j}$	−0.02546	0.006623	0.01426	0.0188	−0.02664	−0.009781	0.009608	0.004847
$Y_{j}$	14.252	14.664	14.754	14.298	12.628	5.836	4.816	4.321
$N_{0}$	1.5995	1.9027	1.4942	1.5889	1.6317	1.4672	1.8316	1.7151
$\alpha_{j}$	−3.2995	0.9087	1.9805	2.4522	−2.7105	−0.2125	0.1422	0.0577
$\eta_{1}$	−0.0697	−0.1476	−0.04	−0.0465	−0.0869	−0.0504	−0.1201	−0.0797
$\eta_{2}$	0.0087	0.0606	−0.0036	0.00017124	0.0150	−0.0126	0.0438	0.0122

**Table 4:** Ray-tracing parameters of the optimized system.

	**1**	**2**	**3**	**4**	**5**	**6**		**7**
**Glass**	**H-K51**	**H-ZF3**	**H-FK71**	**H-ZF6**	**H-ZBAF21**	**H-ZF6**		**H-ZPK1A**
$\varphi_{j}$	−0.005773	0.009655	−0.008114	−0.008616	0.01	−0.003131		0.018
$Y_{j}$	12.637	12.660	12.289	12.447	13.773	14.137		15.779
$N_{0}$	1.5190	1.7069	1.4542	1.7435	1.7151	1.7435		1.6135
$\alpha_{j}$	−0.5836	0.9796	−0.7757	−0.8450	1.2009	−0.3961		2.8370
$\eta_{1}$	−0.0549	−0.0995	−0.0362	−0.1060	−0.0797	−0.1060		−0.0509
$\eta_{2}$	−0.0042	0.0232	−0.0041	0.0291	0.0122	0.0291		−0.0022
	**8**	**9**	**10**	**11**	**12**	**13**	**14**	**15**
**Glass**	**TF3**	**H-ZF72A**	**H-FK61**	**H-ZPK5**	**F7**	**H-QK1**	**H-ZF52**	**H-ZBAF21**
$\varphi_{j}$	−0.021	0.003113	0.013	0.016	−0.023	−0.009421	0.007063	0.005341
*Y_j_*	15.490	15.556	15.581	14.938	13.025	6.211	4.738	4.298
*N*_0_	1.6062	1.9027	1.4942	1.5889	1.6285	1.4672	1.8316	1.7151
$\alpha_{j}$	−3.1897	0.4769	1.9979	2.2602	−2.4701	−0.2301	0.1004	0.0625
$\eta_{1}$	−0.0719	−0.1476	−0.04	−0.0465	−0.0849	−0.0504	−0.1201	−0.0797
$\eta_{2}$	−0.0014	0.0606	−0.0036	0.00017124	0.0163	−0.0126	0.0438	0.0122

**Table 5:** Tolerance Allocation.

Tolerance Items	Value
Radius (fringes)	1
Thickness (mm)	0.01
Decenter X/Y (mm)	0.01
Tilt X/Y (degrees)	0.0042
S + A irregularity (fringes)	0.2
Index of refraction	0.0003
Abbe number (%)	0.5

## Data Availability

The data presented in this study are available on request from the corresponding author. The data are not publicly available due to technical secrets.
